# Leptospiral Pathogenomics

**DOI:** 10.3390/pathogens3020280

**Published:** 2014-04-10

**Authors:** Jason S. Lehmann, Michael A. Matthias, Joseph M. Vinetz, Derrick E. Fouts

**Affiliations:** 1 Division of Infectious Diseases, Department of Medicine, University of California San Diego, School of Medicine, La Jolla, CA 92093-0741, USA; E-Mails: jslehman@ucsd.edu (J.S.L.); mmatthias@ucsd.edu (M.A.M.); jvinetz@ucsd.edu (J.M.V.); 2 Instituto de Medicine Tropical “Alexander von Humboldt”, Department of Cellular and Molecular Sciences, Faculty of Sciences and Laboratory of Research and Development, Universidad Peruana Cayetano Heredia, Lima 100, Peru; E-Mail: joseph.vinetz@upch.pe; 3 J. Craig Venter Institute, Rockville, MD 20850, USA

**Keywords:** *Leptospira*, pathogenomics, virulence, genomics, evolution, taxonomy, molecular epidemiology, systems biology

## Abstract

Leptospirosis, caused by pathogenic spirochetes belonging to the genus *Leptospira*, is a zoonosis with important impacts on human and animal health worldwide. Research on the mechanisms of *Leptospira* pathogenesis has been hindered due to slow growth of infectious strains, poor transformability, and a paucity of genetic tools. As a result of second generation sequencing technologies, there has been an acceleration of leptospiral genome sequencing efforts in the past decade, which has enabled a concomitant increase in functional genomics analyses of *Leptospira* pathogenesis. A pathogenomics approach, by coupling of pan-genomic analysis of multiple isolates with sequencing of experimentally attenuated highly pathogenic *Leptospira*, has resulted in the functional inference of virulence factors. The global *Leptospira* Genome Project supported by the U.S. National Institute of Allergy and Infectious Diseases to which key scientific contributions have been made from the international leptospirosis research community has provided a new roadmap for comprehensive studies of *Leptospira* and leptospirosis well into the future. This review describes functional genomics approaches to apply the data generated by the *Leptospira* Genome Project towards deepening our knowledge of virulence factors of *Leptospira* using the emerging discipline of pathogenomics.

## 1. Introduction

### 1.1. Leptospirosis

Leptospirosis, caused by pathogenic spirochetes belonging to the genus Leptospira, is a zoonosis that has important impacts on human and animal health worldwide [1]. While the exact global disease burden remains unknown, recent estimates by the Leptospirosis Burden Epidemiology Reference Group (LERG) at the World Health Organization have set the number of human cases of severe leptospirosis to over 500,000 per year [2]. This number almost certainly represents an under-representation due to poor surveillance and difficult diagnosis [3]. Clinical symptoms range from a self-resolving acute undifferentiated febrile illness to severe, sometimes fatal disease with renal failure, jaundice, hemorrhage (particularly affecting the lungs), and vascular collapse [4]. Leptospirosis has gained attention worldwide due to recent epidemics of fatal leptospirosis-associated severe pulmonary hemorrhage syndrome without jaundice or renal complications that was originally diagnosed following heavy flooding in rural Nicaragua [5,6]. This severe pulmonary hemorrhage syndrome due to leptospirosis is now recognized as a cause of death worldwide. Transmission to mammals occurs via direct contact with leptospire-infected urine or tissues or indirectly through contact with contaminated soil or water. Although infection may take place through unbroken skin after prolonged immersion, Leptospira usually gain entry to the host via abrasions or cuts in the skin or through exposed mucosae (eyes, nose, *etc.*). Incidence is seasonal, peaking in summer and fall in temperate climates and during the rainy season in tropical areas, mirroring the ability of the bacteria to survive in the external environment. Soil [7,8,9], mud [10], and surface waters [11] contaminated with urine from chronically-infected reservoir hosts remain important sources of human leptospirosis transmission worldwide. Flood-associated epidemics are increasingly reported, ranging globally from Hawaii to the Philippines, to even occasionally continental Europe [12,13,14,15,16]. Whether this is due to improved diagnosis or disease emergence is currently unknown. Clinically apparent disease is more commonly found in urban than rural regions and men have consistently been found to experience more severe disease after infection than women.

The natural host immune response to leptospirosis is mediated largely through humoral mechanisms [17], where protective agglutinating antibodies produced during infection are directed mainly towards leptospiral lipopolysaccharide (LPS). This immunity is protective against a limited number of very closely related homologous serovars. Toll-like receptor TLR2 and TLR4 have also been found to be necessary for effective innate immune control of infection [18]; however, the immunological responses underlying the differences between infection of reservoir and incidental hosts remain a mystery.

Human leptospirosis is more common in developing countries, but globalization and international travel have led to its apparent increased incidence in industrialized countries, usually, but not exclusively, associated with eco-sports, such as white-water rafting and triathalons, as well as military-related activities [19,20,21,22]. Due to limited understanding of leptospiral ecology, disease transmission and pathogenic mechanisms, and a paucity of experimental work to identify and validate factors important for infection or virulence, only limited progress has been made towards implementing effective public health responses. Though killed, whole-cell vaccines are registered for veterinary use (cattle, pig, dogs), no vaccine is registered for humans and challenges in prevention, diagnosis, and clinical management remain.

**Figure 1:**
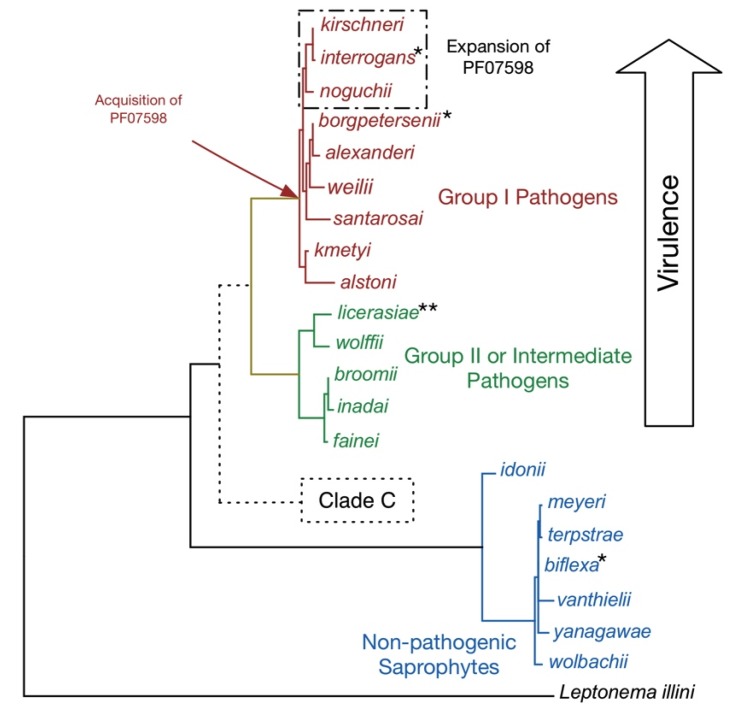
Taxonomy of the Genus *Leptospira*. A phylogenetic tree of full-length 16S rRNA sequences showing the relatedness of the 21 recognized leptospiral species. The putative Clade C has been detected in Peruvian surface waters by qPCR [23] and includes strains/species of unknown pathogenicity. For brevity, the *Leptospira* genus name has been omitted. Key inferred evolutionary events as indicated by whole genome comparisons are shown. Prophages have been detected in *L. interrogans*, *L. licerasiae*, and *L. biflexa*, but not *L. borgpetersenii*. Of these, only in *L. interrogans* have the putatively antiviral “clustered regularly interspaced short palindromic repeats” (CRISPR) elements been identified. By contrast, *L. licerasiae* and *L. biflexa* have an expanded repertoire of type II and type III toxin-antitoxin systems, which could have anti-phage activity. Of the pathogenic species, only the more virulent Group I pathogens have genes for putative virulence proteins belonging to the paralogous family matching Pfam model PF07598, which we suggest help determine tissue-specific colonization. The outgroup is the closely related spirochete *Leptonema* *illini*. For the published genomes, complete genomes are available for six strains of three species (*) and high-quality draft genomes are currently available in GenBank for three strains (**). Genomes representing the remaining *Leptospira* species are available in GenBank (manuscript in preparation).

### 1.2. Leptospira

The genus *Leptospira* includes at least 21 species arranged into three large subgroups based on 16S rRNA phylogeny (Figure 1), DNA-DNA hybridization (until recently the gold-standard for defining bacterial species), pathogenicity, virulence, and *in vitro* growth characteristics. The infectious group (Groups I and II; previously called “pathogens” and “intermediate pathogens”, respectively) includes 14 species (nine in Group I and five in Group II) and the non-infectious group comprised of seven species referred to as “saprophytes”. Group I pathogens [24,25] have been classified into over 250 distinct serotypes and produce disease in people varying in severity, ranging from subclinical infections to severe disease and death; most, if not all, severe disease is caused by serovars belonging to the evolutionarily-related species *L. interrogans*, *L. kirschneri*, and *L. noguchii*. By contrast, Group II pathogens [24,26,27,28,29,30] grow better in culture and cause predominantly mild self-resolving illnesses without fatal complications. Saprophytic *Leptospira* [24] are free-living environmental microorganisms. A new non-infectious species *L. idonii* has recently been described [31] and evidence for another subgroup, designated Clade C, comprising species of unknown pathogenicity has been detected by qPCR in the peruvian Amazon [23].

How and why *Leptospira* evolved from a free-living non-infectious environmental organism is hotly debated. Virulence determinants are poorly understood, as are the mechanisms by which the bacteria produce disease. It is now possible to leverage improved bioinformatics tools to address these and other relevant questions in the field using data from a large leptospiral genome sequencing effort utilizing a hybrid 454/Illumina sequencing strategy (manuscript in preparation) that has provided high-quality draft genomes for representative strains from 20 of the 21 recognized species. “Pathogenomics” approaches coupling high throughput sequencing and bioinformatics comparisons of gene content are indicating specific phenotypic changes that distinguish saprophyte from pathogen. In addition, this approach is resolving questions regarding the pathogenicity of Group II *Leptospira* species (manuscript in preparation).

### 1.3. Maintenance Hosts

*Leptospira* colonize the renal tubules of chronically infected reservoir animals and are shed via urine into the environment. Many mammalian species and amphibians [32,33] may act as reservoirs of *Leptospira*. Some host species are believed to favor specific serovars (e.g., serovar **Copenhageni** in rats, **Lai** in field mice and **Hardjo** in cattle), but these serovar-host associations are not absolute. High frequency infection by non host-adapted serovars has also been documented (e.g., serovar **Ballum** in rats [34]). The expectation that reservoir hosts remain asymptomatic during infection has recently come into question considering infrequent outbreaks caused by serovar **Pomona** that occur among California Sea Lions, which are believed to be reservoirs of this serovar [35]. Perhaps outbreak serovar **Pomona** strains represent emerging genotypes that differ from benign carriage **Pomona** strains at other loci? Whole genome comparisons of these with non-outbreak strains could improve our understanding of pathogen emergence. Whereas reservoirs shed *Leptospira* in urine, often for the lifetime of the animal, in non-reservoir (“incidental”) hosts, such as humans, renal colonization and leptospiruria rarely persist for more than a few months, although chronic renal infections by Group I and Group II strains lasting a year or more have also been documented [36]. Host animals transmit *Leptospira* to other animals through contact with infected urine, via sexual transmission [37], by vertical transmission from infected mother to susceptible offspring [38,39] and probably indirectly, through contact with contaminated water and soil [40,41]. Rats were the first recognized carriers of *Leptospira* [42] and remain an important source of transmission in urban areas [43,44]. Other important reservoirs include domesticated dogs, pigs, cattle and horses, and also wild animals (e.g., the spiny rat (*Proechimys* spp.)) in Latin America and frogs and toads in the Caribbean [32,45]. Curiously, some reservoir hosts for some serovars are also incidental hosts for non-host adapted serovars (e.g., dogs maintain serovar **Canicola**, but often develop severe disease when infected with serovar **Australis** [46] or **Grippotyphosa** [47]). Potentially any vertebrate species can be considered to be susceptible to acute and chronic infection by certain strains, and thus excrete pathogenic *Leptospira* into the environment [48]. This includes marine mammals such as free-living Californian sea lions (*Zalophus californianus*) [35,49,50,51] and northern elephant seals (*Mirounga angustirostris*) [52]. Indeed, new animal sources of carriage, including bats [53], continue to be identified. Host range determinants are unknown, though comparative genomics suggest that the structure of the O-antigen is important. As serovars **Lai** and **Copenhageni**, in which the O-antigen gene clusters are 100% identical at the amino acid level [54], have different reservoir hosts; and some species (e.g., rats [28]) can maintain multiple serovars, structural variation of the O-antigen alone cannot explain host predilection.

### 1.4. Pathogenomics: An Overview

To address the basic question “what makes bacteria pathogenic?” we need to know the functional differences between pathogenic and non-pathogenic strains or species. Early comparative genomics studies, based on Sanger or 454 sequence data, compared up to six isolates of the same or different species [55,56,57,58]. These initial studies focused largely on determining the “core” (*i.e.*, genes found in all strains of a species) *versus* the “variable” genes of the pan-genome, and whether a particular pan-genome was “open” (and amenable to DNA transfer from other strains or species) or “closed” (closed off to genetic exchange) [59,60]. The variable genome is comprised of genes that are strain specific or are found in some but not all strains. As genome plasticity (gain or loss of genes) allows pathogens to adapt to changing environments, studies of dispensable and strain-specific genes can provide valuable insights into virulence and pathogenesis. In addition, mechanisms mediating genome plasticity (e.g., interstrain or interspecies gene transfer, mutation, and selection) will enhance our understanding of the forces shaping microbial evolution and contexts in which they occur.

As a newly emerged discipline, Pathogenomics seeks to delineate virulence factors and their contributions to overall pathogenesis by comparing gene repertoires of pathogenic and non-pathogenic strains/species (reviewed in [61]). With the advent of higher throughput sequencing technologies and improved bioinformatics tools, it is now possible to sequence and compare dozens of strains of the same or different species. As more strains can be sequenced and compared, new comparative studies use these datasets to address more focused questions (e.g., the evolutionary roles of single nucleotide polymorphisms in core and strain-specific genes, lateral gene transfer (LGT), and prophage lysogenization [62,63]). This review introduces some of the available software for comparing microbial genomes, summarizes current *Leptospira* genomics and pathogenomics efforts, and illustrates how these data are being used to better understand leptospiral evolution, pathogenicity, and virulence. Finally, we highlight how these approaches can be leveraged to improve leptospirosis prevention, diagnosis and treatment.

## 2. Software

Several computational tools have been developed to cluster orthologous protein sequences in order to compare predicted functions across multiple isolates (e.g., Sybil [64], OrthoMCL [65], InParanoid [66]). These programs also include paralogs within clusters, but ignore information about expansion and contraction of paralogous protein families, which are important factors in phenotype and genome evolution. They also do not consider genomic neighborhoods of orthologous genes, which can be important for coordinated gene regulation in bacteria through coupled transcription-translation in operons and may give new insights into how genes are acquired and spread. Lastly, none of these programs detect frame-shifted genes, which could represent dispensable genes or sequencing errors. To address these issues, the Pan-Genome Ortholog Clustering Tool (*PanOCT*) was developed [67]. *PanOCT* is a graph-based tool written in PERL that utilizes the BLAST score ratio (BSR) [68], conserved gene neighborhood (CGN) and frame-shift detection in a weighted scoring scheme to generate non-paralogous ortholog gene clusters from multiple bacterial genomes. *PanOCT* was used in a recent publication on the pathogenomics of *L.* *interrogans* [69].

A number of bioinformatics tools have been created to identify putative prophage regions within bacterial genomes [70,71,72,73,74]. *Phage_Finder* automates the once laborious process of prophage finding and is open source. *Phage_Finder* is a heuristic program written in PERL that uses input BLASTP matches to a database of known phage sequences and also to phage-specific Hidden Markov Models (HMMs) to locate regions of the genome enriched with phage-like genes. Using a set of heuristics, each initial region is extended outward based on the annotation of neighboring genes until housekeeping genes are found. Attachment (*att*) sites are specific sequences used for phage integration. *Phage_Finder* is one of the only programs that predict *att* sites, resembling direct repeats, using local alignment algorithms. The site of phage insertion is then predicted based on the location of the predicted *att* sites. Insertion can occur at intergenic sites or within tRNA, tmRNA or protein-coding genes. Due to genetic exchange with other mobile elements, *Phage_Finder* can also identify genomic islands (GIs) and integrated plasmids. In addition to identification of prophage and GIs, *Phage_Finder* can classify regions based on homology to phage family-specific proteins—version 2.0 of the package, which utilizes HMMER3 [75,76], is publicly available [77]. Other computational tools have been developed to predict GIs utilizing either sequence composition bias or comparative genome approaches. One such tool, IslandViewer [78], is a web accessible application that predict GIs using uploaded sequence data [79] combining output from several accurate independently developed methods for GI prediction: IslandPick [80], IslandPath-DIMOB [81] and SIGI-HMM [81]. Whereas most GI prediction tools utilize either sequence composition bias or comparative genomics, IslandViewer integrates both approaches, resulting in enhanced sensitivity and specificity. Data can be retrieved at both the chromosome and gene level for method-specific or consistent GI prediction.

## 3. *Leptospira* Genomics and Pathogenomics Studies

### 3.1. Mobile Genetic Elements

Differences among bacterial strains include mobile DNA elements (e.g., in *Leptospira* include prophages, transposons, insertion sequence (IS) elements, plasmids, genomic islands [55,56,57,58,82,83]). Many of these DNAs encode proteins involved in cell surface structures (*i.e.*, O-antigen, capsular polysaccharides (CPS), teichoic acid, S-layer, flagella, pili, and porins), toxins and/or resource utilization. Regions that vary between strains have been referred to as “flexible” GIs [84] and are of particular interest in microbial studies because these mobile genetic elements (MGEs) may introduce virulence factors into a new host genome.

#### 3.1.1. Bacteriophages

Predation by bacteriophages, which use specific bacterial cell surface structures as receptors, is thought to be a major force driving bacterial evolution. Natural selection by bacteriophage rather than the host immune system could be important in bacterial O-antigen evolution, though this has never been tested in *Leptospira*. Temperate bacteriophages, those that can integrate into the host chromosome, can alter the phenotype of their host (*i.e.*, lysogenic conversion) via delivery of genes involved in adaptation of the host to new environments and can mediate serotype conversion of the O-antigen [85]. Pathogenomics inquiries indicate that in *Leptospira*, lysogenization might have played an important role in leptospiral evolution since prophages, some of which encode proteins with important functions, have been found in representative species from each of the three major branches (Figure 1). For example, in the intermediate pathogen *L. licerasiae*, a novel LE1-like prophage, called vB-LliZ_VAR010-LE1 (using systematic bacteriophage nomenclature [86]), encodes efflux pumps sharing homology with chromosomally encoded proteins in pathogenic species [87]. As these pumps are absent from the saprophyte, *L. biflexa*, they may function in adaptation to the mammalian host. A second prophage region identified in *L. licerasiae* is adjacent to a cryptic prophage (LA0186 to LA0219) expressed in *L. interrogans* serovar **Lai** 56601, which is thought to be associated with pathogenicity since expression is down regulated in the avirulent *L. interrogans* serovar **Lai** IPAV [88]. Of note, although it took several years for the LE1 phage [89,90] and a cryptic prophage region [88,91] to be identified and sequenced, the LE1-like phages were quickly identified using high throughput sequencing and bioinformatics. Comparative approaches also independently identified both LE1 and cryptic prophage regions [87].

#### 3.1.2. Evidence for Plasmid Transfer

Type II and type III toxin-antitoxin systems (TASs) belong to the class of bacterial MGEs as they are extensively, if not preferentially, spread via plasmid-mediated LGT [92]. Several roles have been proposed, including non-functional roles such as “junk DNA” and chromosomal remnants from transposons and bacteriophages, and functional roles including gene regulation [93], programmed cell death [94] and anti-phage activity [95,96]. Like many, if not most MGEs, TASs are not simply mobile, but appear to behave like selfish elements, as they contribute to the stable maintenance and dissemination of plasmids and genomic islands in bacterial populations, seemingly despite associated fitness costs. Of the 28 TASs in the *L. licerasiae* genome, 10 (36%) are associated with putative GIs and a further four are unique amongst *Leptospira*. 37% of these systems are also located on genomic islands in *M. tuberculosis* [97]. Identifying genes associated with these MGEs could provide insight into previously unknown ecological differences amongst *Leptospira*. For instance, three of five putative *L.* *licerasiae* type III TASs are unique to the genus. *L. interrogans* possesses five TASs that are not present in the saprophyte *L. biflexa* [98,99], four of which have not been found in other pathogenic *Leptospira* species; therefore, adjacent genes might provide insight into unique virulence mechanisms. Of the completed genomes, only *L. biflexa* possesses a circular plasmid [100], though a 54-kb GI in **Lai** 56601 has been shown to excise from the chromosome and exist as a plasmid [101].

#### 3.1.3. Repetitive Elements

**Figure 2:**
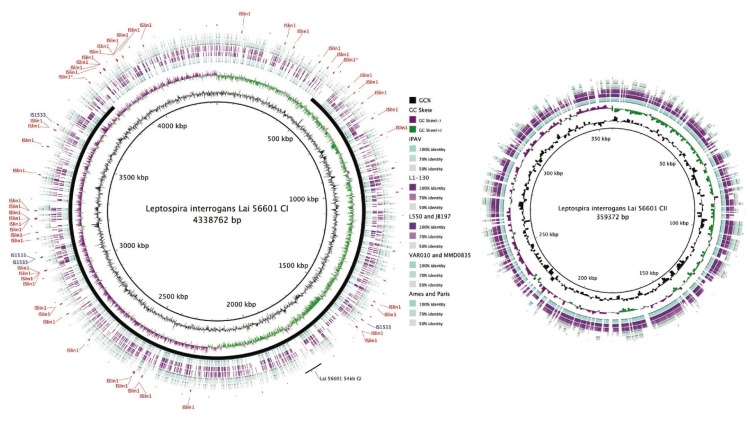
Blast Ring Image Generator (BRIG) [115] plot showing whole genome comparison of *L. interrogans*, *L. borgpetersenii*, *L. licerasiae* and *L. biflexa*. Track 1 (innermost; reference genome): *L. interrogans* **Lai** 56601; Track 2: GC%; Track 3: GC Skew; Track 4: *L. interrogans* **Lai** IPAV; Track 5: *L. interrogans* **Copenhageni** L1-130; Track 6: *L. borgpetersenii* **Hardjo** (JB197 and L550); Group II (Track 7): *L. licerasiae* **Varillal** (VAR010 and MMD0835); and Track 8: *L. biflexa* **Patoc** (Ames and Paris) showing conserved proteins with amino acid similarity of ≥50%. The locations of two common IS elements in **Lai** 56601: ISlin1 (red) and IS1533 (blue) are shown. The solid black line shows the inverted region of the **Lai** 56601 genome (with respect to **Copenhageni** L1-130); the two IS elements believed to have mediated the inversion have been designated with an asterisk (*). The location of 54-kb GI in **Lai** 56601, capable of excising from the chromosome and replicating as a plasmid [101], is shown. Homologous coding regions have been color-coded based on %identity as indicated by the key. All *Leptospira* contain both the CI and CII replicons; whereas only *L. biflexa* contains a large plasmid designated p74 (not shown).

IS elements are short DNA sequences that act as simple transposable elements. They are usually smaller than other transposable elements (700 to 2500 bp in length); and only code for proteins necessary for transposition. Several IS elements have been described in Group I pathogenic *Leptospira*, including IS1500 [102], IS1501 [103], IS1533 [104], and ISLin1 [54]. By contrast, few have been identified in Group II pathogens [87] or saprophytes [100]. Thus, an expansion of IS elements could be a distinguishing feature of Group I pathogens [105]. As these elements are thought to help mediate gene acquisition [106], inactivation or deletion [107], or large-scale genome rearrangements (Figure 2 and [54]) by transposition or homologous recombination, the genomes of Group I pathogenic *Leptospira* may be less stable and perhaps more versatile by comparison. For example, *L. borgpetersenii* serovar **Hardjo** strains L550 and JB197 contain 77 and 84 complete copies of IS1533, respectively, and roughly 25 partial copies distributed throughout each genome, indicative of frequent transposition and recombination events [107]. IS1533 insertion and subsequent recombination has disrupted a *crc*-like gene leading to a 41-kb insertion in *L. borgpetersenii* compared to *L. interrogans*, and in **Lai** 56601, several genes appear to have been inactivated by IS elements compared to **Copenhageni** L1-130 [54]. IS-mediated gene inactivation/deletion may have significant consequences, potentially altering transmission modes. For instance, genome reductions of **Hardjo** L550/JB197 could have caused a diminished capacity of the strains to grow in artificial media [107]. Due to this poor growth *ex vivo*, it has been suggested that these strains are becoming obligate parasites that are likely to be transmitted exclusively from animal to animal [102]. A large inversion, presumably due to recombination between ISlin1 elements, has been documented in **Lai** 56601 [54]. Though the consequences of this inversion are unknown it is unlikely to significantly impact gene expression since the distance of affected genes from the replication fork is unchanged [108,109,110,111,112,113]. IS elements could also facilitate host-range switching. For example, serotype conversion of **Copenhageni** (carried by rats) to **Hardjo** (cattle) is thought to be IS-mediated [114].

#### 3.1.4. O-Antigen Diversity

Host range determinants are unknown as are the mechanisms of cross-species transmission, host range expansion or host shifts, though comparisons of O-antigen (*rfb*) loci are providing important insights. For instance, two genetically distinct, but serologically indistinguishable subtypes of the bovine-adapted serovar, **Hardjo**, have been described: *L. borgpetersenii* serovar **Hardjo** subtype Hardjobovis and *L. interrogans* serovar **Hardjo** subtype Hardjoprajitno. The *rfb* loci of both subtypes share considerable similarity and have been divided into four gene clusters based on sequence similarity to other leptospiral *rfb* loci. Two of the four clusters in subtype Hardjoprajitno are more similar to corresponding gene clusters of subtype Hardjobovis, while two (orfJ15-orfJ20 and orfJ23-orfJ31) are almost identical to gene clusters in the rat-borne *L. interrogans* serovar **Copenhageni** [114]. This suggests that the ancestral subtype Hardjoprajitno strain was most likely a **Copenhageni** strain that acquired orf 1–14 and orf 21–22 from subtype Hardjobovis, resulting in serologically indistinguishable **Hardjo** subtypes. Inspection of these gene clusters (and their encoded functions) could provide important insight into host-range determinants. It’s tempting to speculate that evolutionary pressures such as the aforementioned phage predation that affect O-antigen diversity, whether they occur *in vivo* or *ex vivo*, are likely to affect host range and/or pathogen emergence (e.g., evolution and emergence of El Tor O1 and O139 serotypes in *Vibrio cholera* appear to be phage mediated [116] or a result of large fragment DNA transformation [117]). Disentangling the mechanisms of O-antigen evolution and the contexts in which such evolution occurs could improve our understanding of transmission cycles and mechanisms of serotype emergence.

Sandwiched within the *rfb* locus of *L. interrogans* serovars **Copenhageni** and **Lai** are several genes predicted to be involved in the biosynthesis of the sialic acid, legionaminic acid [118], a known virulence determinant in *Legionella pneumophila* [119]. As sialic acids may have important consequences for survival within macrophages [119] and complement resistance [120], two key leptospiral virulence traits, it could explain why these strains are among the most virulent *Leptospira*. The presence of legionaminic acid—likely by convergent evolution—tempts the speculation that *Leptospira* and *Legionella* may share fundamental features of host adaptation.

**Figure 3:**
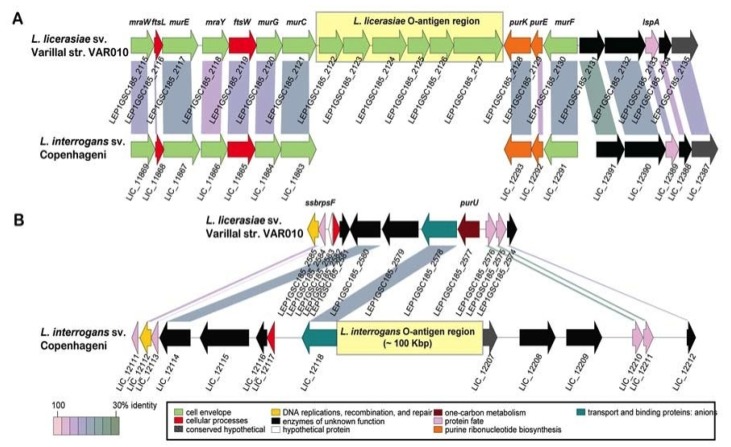
Genetic organization and alternate location of *L. licerasiae* *rfb* locus. (**A**) Comparison of the *rfb* locus of *L. licerasiae* strain VAR010 and homologous region in *L. interrogans* serovar **Copenhageni**; (**B**) Comparison of the *rfb* locus of *L. interrogans* **Copenhageni** and homologous region in *L. licerasiae* strain VAR10. Yellow shaded boxes mark the locations of the O-antigen regions. CDSs are labeled by locus identifier and colored by functional role categories as noted in the boxed key. Gene symbols, when present, are noted above their respective genes in bold italics. Reproduced from [87].

Compared to other *Leptospira*, the *L. licerasiae* O-antigen biosynthesis region is unusual, consisting of a short six-gene cassette [87] that includes three glycosyltransferases inserted between two normally adjacent, convergently transcribed genes: the *murC* gene of cell wall biosynthesis and *purK* gene of purine biosynthesis. Indeed, the glycosyltransferase, LEP1GSC185_2122 (GenBank: |EIE02925) in *L. licerasiae* str. VAR010, serves as a good marker for absence of the typical (long) O-antigen biosynthesis region and replacement by a six-gene cassette between *murC* and *purK* (Figure 3). Genes in these regions have no close homologs in any other *Leptospira*, in the O-antigen region or anywhere else, supporting the notion that these cassettes were acquired by LGT and provide unique carbohydrate chemistry and serology. These and other less dramatic differences in *rfb* loci underlying serological diversity present an opportunity for development of DNA-based typing tools for serological classification that could replace the tedious and sometimes unreliable cross-adsorption agglutination reactions traditionally used to serotype *Leptospira* [121].

The reasons for the unusual arrangement of the *L. licerasiae* *rfb* locus are unknown, but could underpin key differences in host adaptation or other ecological relationships between this and other leptospiral species.

### 3.2. Virulence Mechanisms and Pathogenesis

Pathogens possess a number of virulence factors, and delineating and understanding the function of these determinants is paramount to understanding the pathogenesis of the diseases they cause. The combinatorial effect of these factors enables microbes to efficiently invade and colonize various tissue niches, obtain nutrients, as well as evade and suppress the immune response of the host. In contrast to other pathogens, where experimental genetic inquiry has defined a number of virulence factors, the mechanisms by which pathogenic Leptospira cause disease remain largely unknown; mostly due to the recalcitrance of pathogenic Leptospira to genetic manipulation. There have been reports of site-directed homologous recombination being used for the deletion of chromosomal genes; however, these reports are exceptions rather than the norm. Targeted gene knockouts still remain out of grasp for most Leptospira researchers; and no replicative plasmid vector is available for pathogenic Leptospira. Alternative approaches to discover virulence related genes in Leptospira have included evaluating transcriptional responses during exposure to “host-like” conditions *in vitro* [122,123,124,125,126,127]; however, global gene regulation in response to the combination of all extracellular cues *in vivo* remains to be investigated. The known leptospiral virulence factors have been extensively reviewed [128,129,130], and include LPS (a general virulence factor of Gram-negative bacteria), flagella, heme-oxygenase, the OmpA-like Loa22, and adhesion molecules. In addition, hemolysins and sphingomyelinases may play a role during infection, although there are conflicting reports regarding their true contributions to overall virulence [130].

#### 3.2.1. Genetic Manipulation

As targeted knockouts remain difficult, genetic manipulation of pathogenic *Leptospira* has been limited to random transposon-based mutagenesis in two of the more virulent serovars **Lai** [131] and **Manilae** [132]. This approach has led to the generation of mutant libraries that can then be screened for defects in pathogenesis, and in fact has identified many of the limited number of leptospiral virulence factors known [122,133,134,135]. However, identifying attenuated mutants requires a large commitment of time and resources, and represents the major drawback of this approach. As currently designed, these gene knockout experiments qualitatively (+/– colonization, fatal/non-fatal, *etc.*) evaluate the involvement of a particular gene towards virulence. Comparisons of knockout mutants to wild-type parental strains are by definition binary assessments. While of great value for characterizing the involvement of single determinants to virulence, this approach cannot determine the relative contributions of multiple genes to the total virulence phenotype. Pathogenomic-based approaches provide an unbiased global view of genome-level differences among pathogenic and non-pathogenic strains that can be used as a complementary alternative to transposon-mediated mutagenesis studies.

#### 3.2.2. Attenuation Studies in *L. interrogans* Serovar **Lai**

The genome of a virulence-attenuated *L. interrogans* serovar **Lai** strain IPAV derived by prolonged laboratory passage from a highly virulent ancestral strain was recently sequenced and annotated [105]. Comparisons of this strain and a virulent related strain, *L. interrogans* serovar **Lai** strain 56601, showed a mostly conserved genome (structure and gene order), but identified 33 insertions, 53 deletions and 301 single-nucleotide variations affecting 101 genes in strain IPAV compared to strain 56601 [105]. Genes involved in signal transduction, stress response, transmembrane transport and nitrogen metabolism comprised the majority of the 44 affected functionally annotated genes. Subsequent comparative proteomic analysis of 1627 orthologs revealed that 174 genes in strain IPAV were upregulated *in vitro*, with enrichment mainly in energy production and lipid metabolism functions. By contrast, 228 strain 56601 genes, primarily involved in protein translation and DNA replication/repair, were upregulated compared to strain IPAV [105]. As protein expression comparisons were done *in vitro*, these data provide limited insight into the relevance of these genes during mammalian infection. Moreover, since the ancestral strain was not used in these analyses, it is not clear which of the many differences observed between the two strains resulted in the attenuation phenotype complicating future mechanistic studies. Despite these limitations, this study illustrated that altered expression or mutations in critical genes could account for virulence attenuation in strain IPAV.

In long-term *in vitro* attenuation studies, the only factors likely to be under selective pressure are resource utilization, proliferation and survival in culture media. As virulence associated genes are dispensable *in vitro*, these genes or their regulatory systems could be lost during long-term *in vitro* culture. If stocks are kept of each serial passage (generation), it becomes possible to test the relevance of individual mutations by comparing generations immediately preceding the appearance of the mutation and generations in which the mutation first appeared. A more recent study used this type of study design to identify candidate virulence genes by genomic comparison of a culture-attenuated serovar **Lai** strain 56601_p18 with its virulent, isogenic parent strain 56601_p1 [69]. Of significance, among the genes inactivated by serial *in vitro* passage were several previously unstudied putative virulence genes. The first, a predicted adenylate guanylate cyclase (LA_4008), was found to elevate cyclic AMP activity of *in vitro* cultured monocytes [69]. Increases in the intracellular concentration of cAMP in innate immune cells have been shown to impair phagocytosis, free radical generation, as well as promote the release of anti-inflammatory cytokines. Although modulation of host cAMP levels has been long accepted as a mechanism used by other pathogens to blunt the immune response [136], this is the first evidence of a likely contributor to virulence for *Leptospira*. This is especially important considering evidence that persistence within and killing of macrophages once phagocytosed might be a fundamental difference between reservoir and human macrophages with important implications for the evolution of disease [137].

LA_1056, a hypothetical protein of unknown function, was also identified in our attenuation study. *In silico* sequence analysis revealed a phage tail protein (PHA00965 conserved domain) that shares homology with the phage tape measure protein *pblA* from *Streptococcus mitis*. Studies exploring *pblA* in the pathogenesis of *S. mitis* have demonstrated that bacteria can co-opt tape measure proteins to act as adhesion-type molecules. Specifically, *S. mitis* decorates their outer surface with PblA and use it to bind *α*2-8 linked sialic acid residues on platelet membrane gangliosides [138,139,140]. Thrombocytopenia is a well-documented complication of severe leptospirosis. A prospective study from Barbados found thrombocytopenia in more than 50% of patients hospitalized with the disease, while studies from Brazil found rates of 65% in children and 86% in adults [141,142,143]. Despite this high prevalence, the exact pathogenic mechanisms behind it remain unclear. It is possible that *Leptospira*, through mechanisms similar to those found in *S. mitis*, employ phage-derived proteins like LA_1056 for use as adhesins for attachment to platelets. These data shed light on a poorly understood pathogenic mechanism of leptospirosis that needs experimental validation.

**Figure 4:**
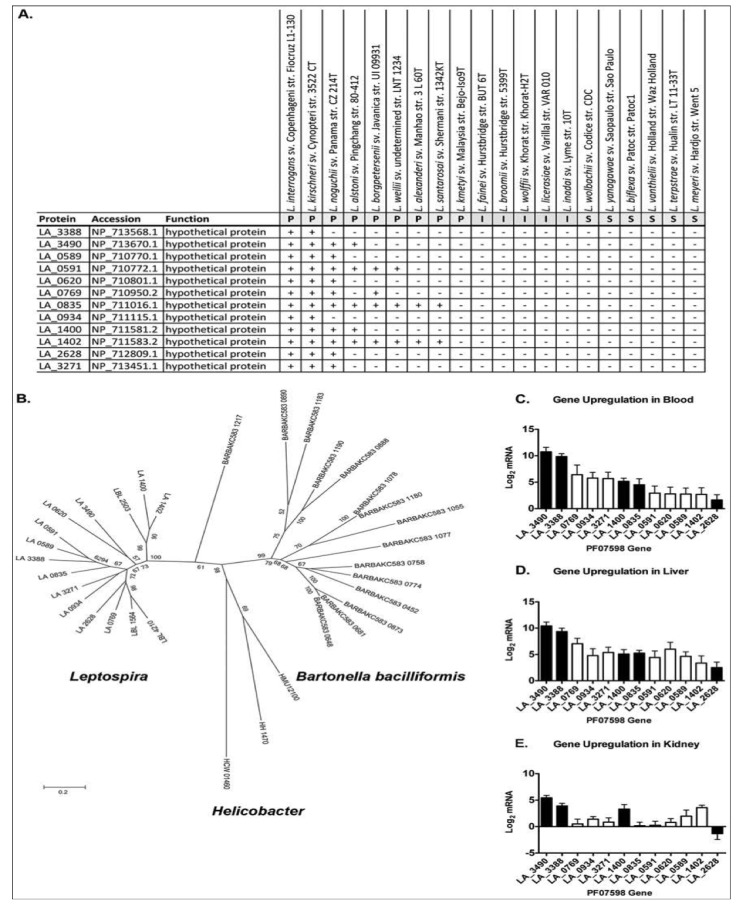
Distribution and expression profile of PF07598 gene family (encoding putatively named virulence modulating or VM proteins). (**A**) Distribution of PF07598 across the genus *Leptospira*; P, pathogen; I, intermediate; S, saprophyte. (**B**) Unrooted phylogenetic tree of the VM protein family including sequences from *Helicobacter* spp. and *Bartonella bacilliformis*. Node labels represent support from 500 bootstrap replicates. (**C**–**E**) Transcript levels of each PF07598 paralog were assessed by real time, reverse transcriptase quantitative PCR of blood, liver and kidney extracts four days after hamster infection and compared to log phase *in vitro* cultured *Leptospira*; expressed as the log2 of the fold change between the two conditions. Solid bars indicate proteins potentially extracellular proteins. Data represented are the mean ± SEM of three independent experiments (n = 7 animals). Reproduced from reference [69].

A fascinating discovery that emerged from the same study was the identification of two virulence-associated genes belonging to a paralogous (PF07598) gene family shared by pathogenic *Leptospira*, but absent in Group I pathogens and saprophytic species (Figure 4A). Paralogs exist in all Group I pathogenic species and are highly up-regulated during infection (Figure 4C,D and [69]). Paralog counts vary amongst the species, ranging from 2 in *L. santarosai* to 12 in *L. interrogans* and *L. kirschneri.* Experimental evidence suggests that members of these proteins may be important for kidney colonization, as a genetic knockout of a PF07598 family member in *L. interrogans* led to reduced renal colonization [144], suggesting that PF07598 paralogs are not functionally redundant. This hypothesis is supported by the fact that different paralogs are differentially expressed in blood, liver and kidney (Figure 3C–E). Why these proteins are absent from Group II pathogens is unknown, but could explain their reduced capacity to cause symptomatic infections. Curiously, PF07598 protein family has been found in the unrelated α-proteobacteria species: *Bartonella bacilliformis* and *B. australis*. *B. bacilliformis* has 15 paralogs and *B. australis* has nearly the same [145]. Single gene copies were also found in three animal-infecting ε-proteobacteria: *Helicobacter hepaticus*, *H. mustelae*, and *H. cetorum*. The reasons for the expansion of the family in the related *L. interrogans*, *L. kirshcneri*, and *L. noguchii* species and *B. bacilliformis* and *B. australis* is unknown, but could indicate a common pathogenic mechanism or larger scale mechanisms of mammalian host/transmission adaptations.

Despite the amount of useful data derived from the study, it was not without its limitations. Notably, unlike the previous attenuation study, this study did not determine gene expression of derivative strains, focused on only protein coding genes and because the genomes were not closed, could not report on the impact of genomic rearrangements *in vitro. In vitro* attenuation has been the basis for many viral and bacterial vaccines (e.g., Yellow fever, measles, mumps, rubella, polio, Bacillus Calmette Guerin (BCG; *Mycobacterium bovis*)). Why is there gene functional loss and mutation during serial passage *in vitro*? Are virulence genes superfluous compared to the energy cost of maintaining expression of these genes during a stage of the organism where pathogen-host interactions are not present, or do expressed virulence genes have a directly toxic effect on the organism?

#### 3.2.3. Comparisons of *L. borgpetersenii*, *L. interrogans*, *L. licerasiae*, and *L. biflexa*

Recent studies underscore the importance of pathogenomics to the study of leptospiral evolution [87,100]. Initial comparisons of *L. borgpetersenii*, *L. interrogans* and *L. biflexa* suggested that infectious species evolved from a non-infectious “free-living” spirochete. As the genome of *L. biflexa* has fewer transposable elements, it is believed to be much more stable than are the genomes of Group I pathogens, which appear to undergo frequent rearrangements, often involving recombination between insertion sequences. For example, significant differences in organization of the *L. borgpetersenii* and *L. interrogans* genomes appear to be IS-mediated. Whereas most putative operons are intact, their order has been shuffled in *L. borgpetersenii* compared to *L. interrogans* [107]. Comparisons of the gene repertoires of *L. interrogans* **Lai** and **Copenhageni** and *L. borgpetersenii* **Hardjo** indicate that genes absent from *L. borgpetersenii* include those involved in signal transduction, which could impair adaptation to and thus survival in, diverse environments [107]. In addition, diminished metabolic capacity and reduced solute transport functions in *L. borgpetersenii* (relative to *L. interrogans*) would likely limit the range of nutrients that can be utilized by *L. borgpetersenii*. By contrast, *L. interrogans* has more signal transduction systems, transcriptional regulatory factors, and metabolic and solute transport functions, consistent with its improved survival *ex vivo*. These data suggest that *L. borgpetersenii* has a reduced ability to survive outside a mammalian host and is likely to be restricted to direct animal-animal transmission, whereas *L. interrogans* has retained environmental sensory functions that facilitate disease transmission through water [107].

**Figure 5:**
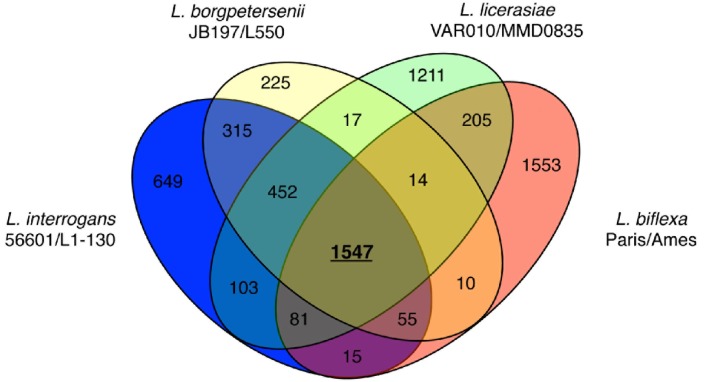
Venn diagram showing the distribution of shared and unique proteins separated by leptospiral species. Two strains from each species were used for these comparisons. Only proteins present in both strains of a given species are shown. Reproduced from [87].

More recent comparisons of eight genomes: *L. borgpetersenii* (2), *L. interrogans* (2), *L. licerasiae* (2) and *L. biflexa* (2) have extended these early observations, deepening our understanding of leptospiral evolution. The existence of a core leptospiral genome comprising 1547 genes—reduced from the 2052 originally reported based on three strains; 452 conserved genes restricted to pathogenic species and likely to be pathogenicity-related (Figure 5); 649 genes in *L. interrogans* that could enhance understanding of virulence mechanisms; and 103 genes common to *L. interrogans* and *L. licerasiae*, but absent from *L. borgpetersenii* that could improve understanding of *Leptospira* environmental sensing and signal transduction systems. Comparisons of the functional content of the genomes suggests that Group II pathogens retain several proteins related to nitrogen, amino acid, and carbohydrate metabolism, which might help to explain why these species grow well in artificial media compared with pathogenic species. Furthermore, several putative GIs are present, suggestive of antecedent LGT with implications for *in vivo* growth and host preference. Indeed, a 54-kb GI in **Lai** (Figure 2) could explain altered virulence characteristics and host preference when compared to **Copenhageni**. How *Leptospira* became naturally competent for transformation remains to be determined, but considering the phylogenetic origins of the genes comprising the O-antigen cluster and other putative laterally transferred genes, *L. licerasiae*—and perhaps other pathogenic strains—must be able to exchange genetic material with non-invasive environmental bacteria. Based on predicted functional content and amino acid sequence identity (Figure 2), these results also demonstrated that Group II *Leptospira* are more closely related genetically to pathogenic than to saprophytic *Leptospira*. The diminished ability of Group II pathogens to colonize or cause disease in mammals compared to Group I may indicate these strains are adapted to non-mammalian reservoir hosts, but this hypothesis has not been formally tested.

### 3.3. Vaccine Candidates

In addition to providing insight into leptospiral virulence and pathogenic mechanisms by connecting clinical metadata, animal model studies and genomic data, pathogenomics approaches have identified several universal anti-leptospirosis vaccine candidates. Despite decades of research, there is still no licensed human vaccine; and veterinary vaccines provide only transient protection. Livestock must be revaccinated every 6–12 months, which is unrealistic in low-income endemic regions. A hitherto insurmountable problem confronting human or veterinary vaccine development arises from the large geographic differences in transmitted serovars. Dominant serotypes in China and India are not found in Europe or The Americas; and most livestock-infecting strains in the US are uncommon in South America.

The pathogen pan-genome is staggeringly large, comprising some 13,000 predicted genes, of which only ~1500 are part of the core leptospiral genome. Pathogenic species can possess between 200–1,200 species-specific genes [87]. In addition, some genes are conserved in specific, but not all pathogenic species, some are sporadically distributed, and some are restricted to only a few closely related species. Thus, it has been exceedingly difficult to identify broadly reacting leptospiral-specific antigens.

Recent pathogenomics inquiries have narrowed the list of potential candidates considerably. The 452 conserved pathogen-specific genes (Figure 5) include LigB, LipL32, LipL41, four LipL45/FecR-related proteins and LruB. LigA and LigB, which are involved in leptospiral adhesion to extracellular matrix proteins and plasma proteins, including collagens I and IV, laminin, fibronectin, and fibrinogen are induced at physiological osmolarity [146]. Other conserved pathogen-specific outer membrane proteins predicted to mediate attachment to host cells include a putative fibronectin-binding protein, Lfb1 [147] and Lsa66 for Leptospiral surface adhesin of 66 kDa shown to bind laminin and plasma fibronectin [148]. As fibronectin-binding proteins are important adhesins that play an important role in certain bacterial infections, these widely conserved pathogen-specific antigens represent important vaccine candidates. Recent experiments in a hamster model have demonstrated that LigB is a valuable protective antigen that could be used in future subunit anti-leptospirosis vaccines [149]. The function of LruB is unknown, but serology suggests this protein is expressed *in vivo* [150]. As more infectious and non-infectious species and strains are compared, this list will be winnowed further. Prioritizing these or secreted proteins will result in several good potential candidates.

### 3.4. Metabolism

As in other bacteria, the availability of different nutrients inside and outside the mammalian host may select for changes in the metabolic capacity of *Leptospira*. Pathogenomics can provide insight into the unique metabolic capacity of pathogenic microbes. This is important for two reasons: the design of improved growth media and novel therapeutics. Pathogenic strains appear to have developed several strategies to obtain essential nutrients *in vivo*. For example, LIC13209, which encodes bacterioferritin-associated ferridoxin (BFD), is conserved among infectious species and is upregulated at physiological osmolarity [127]. The function of BFD is not known, but it may be a general redox and/or regulatory component involved in iron storage or mobilization functions of bacterioferritin in bacteria. Iron is essential for bacterial pathogenesis and is required for processes ranging from the tricarboxylic acid cycle to electron transport, DNA metabolism, and response to oxidative stress; however, iron is poorly soluble at physiological pH in the presence of oxygen and not readily bioavailable, especially as it is sequestered from pathogenic organisms as an innate immune mechanism. Thus, during infection, increased expression of this protein might be required to mobilize cellular iron stores to provide a source of iron, which is present in growth-limiting amounts in the host. Similarly pathogenomics analyses suggest that, unlike the saprophytes, pathogenic *Leptospira* are able to synthesize the essential nutrient B_12_ [87]; though this has not been tested experimentally. Three genes involved in glutathione metabolism are putatively associated with pathogenicity based on comparisons with other spirochetes. In the oral spirochete, *T*. *denticola*, glutathione metabolism is believed to play a significant role in colonization [90]. The potential biological significance of glutathione metabolism by infectious *Leptospira* may be twofold: H_2_S production may be critical for hemoxidative, hemolytic, and other toxic activities that could occur *in vivo* [90], and pyruvate and L-Glutamate, products of glutathione metabolism, can be utilized as nutrients to support bacterial growth and for the synthesis of the essential cofactor vitamin B_12_, respectively.

## 4. Future Directions

Leptospirosis, caused by more than 250 different serovars of the genus *Leptospira*, is the most common and widespread zoonotic disease worldwide. Infection is primarily spread through contact with water contaminated by urine of infected carrier animals. Leptospirosis is clearly an emerging and reemerging infectious disease. There are newly discovered leptospirosis-related syndromes (pulmonary hemorrhage) and newly discovered species of *Leptospira* that cause human leptospirosis. Accessible and geographically useful leptospirosis diagnostics remain unavailable to diagnose disease efficiently, which prevents the accurate assessment of the burden of disease. Obtaining whole genome sequences of a diverse and representative set of globally-significant *Leptospira* is a major priority of the leptospirosis community that will directly facilitate these goals of improving public health through the judicious and well-considered application of fundamental scientific discovery. Future directions of *Leptospira* genome sequencing will include the following:

- To obtain whole genome information for all known *Leptospira* species. Currently there are nine named pathogenic *Leptospira* species, five intermediate *Leptospira* species, and six saprophytic *Leptospira* species; more have recently been informally reported and inevitably these numbers will grow over coming years. This information will provide the basis for identifying a minimal number of molecular markers for multilocus sequence typing that can differentiate infecting leptospires directly from human samples without the need for bacterial isolation. Conserved protein markers that are the targets of antibody recognition or antigen detection will be identified. Accomplishing these goals depends on obtaining whole genome sequence of a globally diverse and representative set of *Leptospira* strains.
- To delineate taxonomic and phylogenetic relationships among *Leptospira* species. Current methods to classify new species or serovars and to identify the emergence of new leptospiral causes of human disease are cumbersome and insufficiently informative. Genomic-level information will allow us to determine serovar without the need for serological typing, will provide fresh insights into the utility of serovar as a tool for strain identification, and if shown to be robust, will facilitate the development of molecular-based serovar typing. Complete genome sequence of reference strains used for serological diagnosis is critical for refining and optimizing efficient diagnosis.
- To understand the mechanisms of leptospirosis pathogenesis and determinants of clinical outcome. Correlations of genetic polymorphisms and virulence will be identified between isolates at the species, serovar, and strain level. This will require sequencing of isolates associated with distinct clinical presentations and outcomes. This data will provide the fundamental basis for hypothesis-driven research to determine virulence factors and for vaccine development.

Key to these efforts will be using pathogenomics tools to comprehensively study *Leptospira* with the following major goals: understanding the global diversity of *Leptospira* and the differences in biological behavior of members of this genus; and determining the sequence of geographically and molecularly diverse set of isolates that represent all current *Leptospira* spp. and diverse serovars. Key outcomes will include a true phylogenetic picture based on global isolates and whole genome data that will lend new insights into taxonomy, evolution and possible global phylogeographic trends. This information will also aid in selection of appropriate targets for diagnostic assays, vaccine development and the development of tools for molecular epidemiology studies.
